# Disseminating “hidden” scientific collections: the medium and large-sized terrestrial mammals at the Museo di Anatomia Comparata “Giovanni Battista Grassi”, Roma, Italy

**DOI:** 10.3897/BDJ.12.e124810

**Published:** 2024-07-05

**Authors:** Alexandra M. R. Bezerra, Edoardo Di Russo, Riccardo Castiglia

**Affiliations:** 1 Museu Paraense Emilio Goeldi, Belém, Brazil Museu Paraense Emilio Goeldi Belém Brazil; 2 Fiocruz/IOC, Rio de janeiro, Brazil Fiocruz/IOC Rio de janeiro Brazil; 3 Dipartimento Biologia e Biotecnologie "Charles Darwin", Università di Roma "La Sapienza", Rome, Italy Dipartimento Biologia e Biotecnologie "Charles Darwin", Università di Roma "La Sapienza" Rome Italy; 4 Istituto Scienze Marine ISMAR, Consiglio Nazionale delle Ricerche CNR, Venice, Italy Istituto Scienze Marine ISMAR, Consiglio Nazionale delle Ricerche CNR Venice Italy

**Keywords:** 17^th^ century, natural history collection, scientific disclosure, threatened species

## Abstract

The dissemination of specimen data in scientific collections is a crucial step in making them available to the scientific community. However, even today, especially in some countries, little or nothing is known about the contents of the naturalistic collections of some museums. This is regrettable, especially in cases where the collections include historic specimens and endangered species. The Museum of Comparative Anatomy “Giovanni Battista Grassi”, situated in Rome, Italy, houses historical anatomical and didactic collections, with specimens gathered from 1600s and almost worldwide. The collection holds 444 specimens of medium and large-sized terrestrial mammals, comprising 25 fossils, 40 skins, 186 skulls, 70 skeletons and 123 anatomical pieces, representing 63% of recent mammal orders, mainly from localities of Africa and Europe. A list of this material, indexed by the orders and families, is provided, as well as comments on the conservation status of the species. Remarkable data are summarised, including new data on a hippopotamus specimen from an extinct population and the record of three rhinoceros species from 1600s. Besides comparative anatomical studies, the Museum of Comparative Anatomy of Sapienza University emerges as a source of important material for biodiversity genomics.

## Introduction

The effort to disseminate specimen data in scientific collections is an important step in making them known to the scientific community (Colella et al. 2021, Cilli et al. 2023). Examples include databases of holdings from scientific collections made digitally available on the World Wide Web through the online facilities, such as GBIF (GBIF 2024), SpeciesLink (SpeciesLink 2002) and VertNet (VertNet 2016). However, much of these data is sometimes not revised, i.e. it is directly replicated from the catalogue book. Some attempts to solve this issue are the selected catalogues of specimens published by curators and/or expert taxonomists (e.g. Bezerra et al. 2004, Calvini et al. 2016 Bezerra and Bordallo 2018, Nascimento and Vendramel 2020, Serrano-Villavicencio et al. 2020, Ghiraldi et al. 2021, Lo Brutto et al. 2023), which provide revised and detailed information on one or more taxa, generally at the order and family levels.

The Museum of Comparative Anatomy “Giovanni Battista Grassi” (hereinafter referred as the Museum), currently located at the Biology and Biotechnology Charles Darwin Department of “La Sapienza” University of Rome, Italy, includes collections obtained from the 17th century until today (Gippoliti and Castiglia 2020, Di Russo et al. 2024). The present museum collection and location are derived from a complex history. Founded in 1873, the collection was handed down from the Mineralogia et Historia Naturalis Museum at the “Pontificio Romano Archiginnasio”, whose first setting dates back to 1805 (Giuseppini and Capanna 2010, Anonymous 2015). Some of the specimens are even more ancient, as they arrived at the Archiginnasio from the collections established at the Roman College by the German Jesuit Attanasio Kircher (1602-1680) (Merzagora and Capanna 2001). After the dismantling of the Archiginnasio, the Museum was initially located in an ancient convent; then, around 1930, it was transferred to its current location (Gippoliti and Castiglia 2020, Di Russo et al. 2024). Since then, the collection has grown with new acquisitions, most of specimens aiming for didactic purposes, under the responsibility of the evolutionist professor Giovanni Battista Grassi (1854-1925), after whom the Museum is named. In 1979, the present Museum was established as an independent structure and improvements and expansions began, gradually making it possible to acquire new exhibition spaces, laboratories and warehouses (Anonymous 2015).

The Museum currently includes about 14,000 samples, representative of all higher vertebrate taxa, being around 6,000 composed of representatives from 22 mammalian orders (Di Russo et al. 2024). Mammals have a worldwide distribution and account for about 6,500 species currently recognised (Burgin et al. 2018). This number has increased in the last two decades, mainly due to new taxonomic discoveries and occurrence records, based on specimens housed in scientific collections (e.g. Feijó et al. (2018), Roos et al. (2020), Semedo et al. (2020)). Despite the new discoveries, 85 mammal species have recently become extinct (last 1500 years – IUCN 2023) or are going to become extinct (Rondinini and Visconti 2015, Mendes Pontes et al. 2016, Ceballos et al. 2017). The only testimonials for some of these locally extinct species or populations are housed in scientific collections (e.g. Bezerra (2011), Németh et al. (2017), Roycroft et al. (2021)), denoting a fundamental source of historical DNA (Nakahama 2021, Pasino et al. 2023).

Here, we present an account of the medium and large-sized terrestrial mammals at the Museum of Comparative Anatomy “Giovanni Battista Grassi”, which are prepared for exhibition display, didactic and scientific purposes. Previous efforts to disclose the mammal specimens housed in this useum come from the catalogue of Cetacea by Maio et al. (2014) and contributions on primates and Neotropical mammals (Bruner and Gippoliti 2006, Gippoliti and Castiglia 2020). The present account includes the number of specimens by family, preservation type, type of object and locality (when available). We also comment on conservation status and other remarkable data.

## Material and methods

As defined here, we have excluded the orders Chiroptera, Didelphimorphia, Eulipotyphla, Lagomorpha and Rodentia, which will be addressed in a species-level catalogue after taxonomic specialist identification. The nomenclature follows Burgin et al. (2018) and updates on Mammal Diversity Database (2024), geographic distribution (Marsh et al. 2022) and biogeographic provinces (Udvardy 1975). Threatened status classification follows the IUCN Red List (IUCN 2023), including the categories Vulnerable – VU, Endangered – EN, Critically Endangered – CR, Extinct – EX; additionally, we have used Near Threatened – NT, since some populations may qualify for a threatened category in the near future. The following were also considered: the Convention on International Trade in Endangered Species of Wild Fauna and Fauna (CITES) Appendices I – threatened with extinction risk, II – not necessarily now threatened with extinction, but that may become so unless trade is closely controlled or species whose species in trade look like those listed for conservations reasons and III – a list of species included at the request of a Party that already regulates trade in the species and that needs the cooperation of other countries to prevent unsustainable or illegal exploitation (CITES – Convention on International Trade in Endangered Species of Wild Fauna and Flora 2023).

Data on locality, collector, collection date, sex, preparation type and any other kind of information (such as, if the skull and/or skin is damaged) were included in a spreadsheet Excel® and summarised in table and graphics. Due to the complex history of the collections (including specimens from zoological gardens or donations with no available origin), only a few specimens have available data on the collection locality or collector. The specimens were identified, based on direct comparison with other specimens previously identified by taxonomists and specific literature.

### Collection management

When necessary, original labels were repaired and/or enveloped with protective plastic and worn label lines were replaced. All specimens were catalogued and ‘mapped’ in an Excel spreadsheet, in a manner to mirror the arrangement inside the cabinets. The museum cabinets are organised with three exhibition rooms and one corridor, while another five rooms are dedicated to the collections. Each room is named differently, as are the cabinets within the rooms and the drawers within the cabinets.

Due to educational purposes, some specimens may have been dismembered in the past and displayed in different display cases. We have tried to minimise this problem by reconstructing the identity of each single individual during the listing of the specimens and vouchers, but this bias may still be partly present.

### Data resources

The Museum is accessible through GBIF at the https://scientific-collections.gbif.org/institution/cb7d2ed6-13b4-4a04-ba14-f88ca9ef94d8 and the data from the medium and large-sized terrestrial mammals at the https://registry.gbif.org/collection/61a49292-9dd9-4746-89cf-21f6aad9435e.

## Results

**Figure 1.**
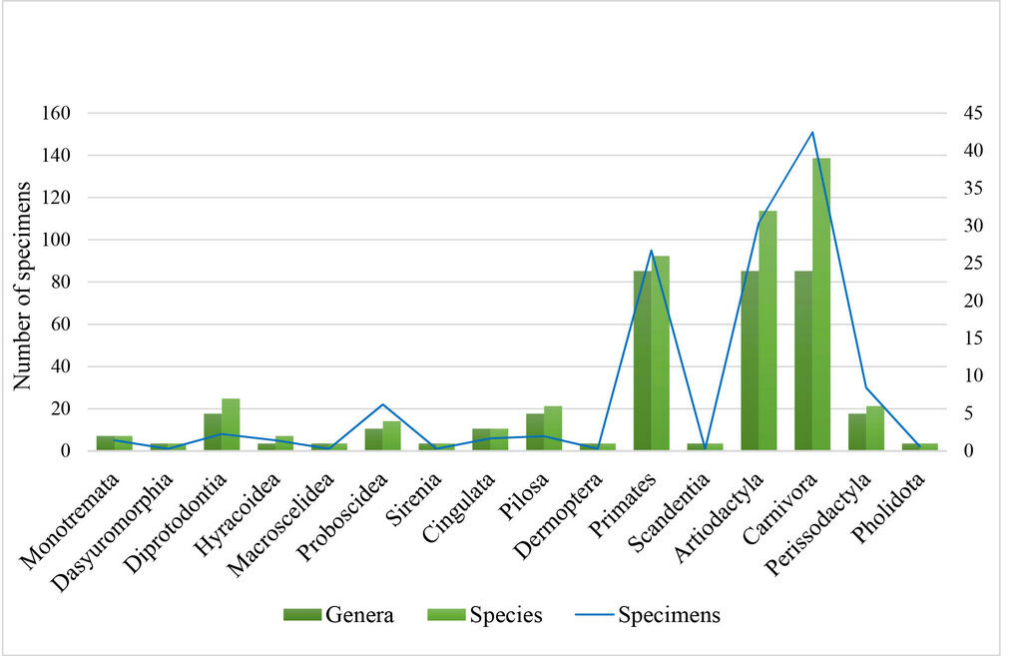
Representation of genera, species and specimens of medium and large-sized terrestrial mammal specimens in the collection of Museo di Anatomia Comparata “Giovanni Battista Grassi” in absolute numbers. Right axis refers to the number of genera and species.

**Figure 2.**
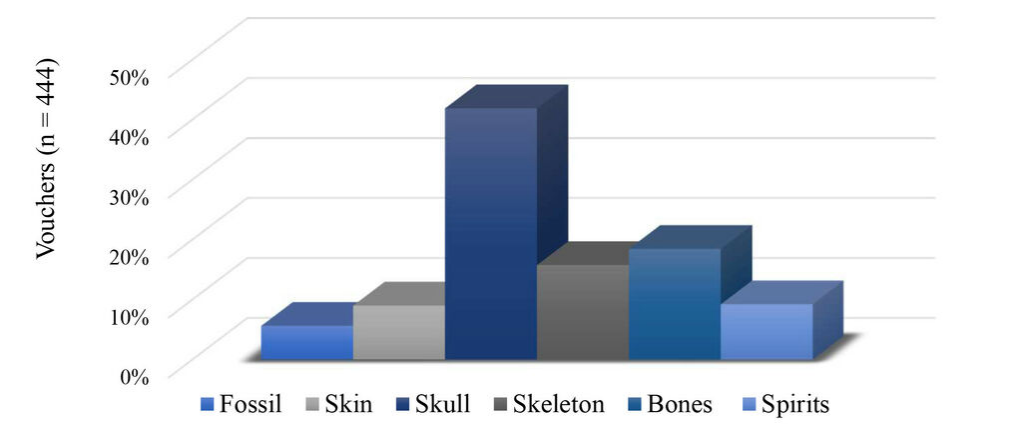
Proportion (%) of medium and large-sized terrestrial mammal specimens in the collection of Museo di Anatomia Comparata “Giovanni Battista Grassi” per preparation type. Skin: stuffed skins for scientific or exhibition proposal; Spirits: whole specimen or parts preserved in 70% ethanol or formaldehyde.

Medium and large-sized terrestrial mammal specimens in the Museum are represented by 444 specimens, including 25 fossils, distributed in 133 species, 16 orders and 50 families from every terrestrial ecoregion (Table 1, Fig. 1, Suppl. material 1). This collection comprises 39 stuffed skins, one open skin, 186 skulls, 70 whole skeletons (hereafter referred as ‘skeletons’), 82 bones and structures, such as vertebra, horns, antlers, teeth and pelvic girdle, and 41 whole and body pieces preserved in spirits, such as tongue, stomach and hearts (summarised data in Fig. 2 and detailed in Suppl. material 1). Due to some anatomical structures being difficult to identify at the genus or family level, they were labelled as ‘Not identified’ and account for 53 anatomical pieces attributed to the marsupial order Diprotodontia and to the placental orders Proboscidea, Primates, Artiodactyla, Carnivora and Perissodactyla (Table 1).

Remarkable taxa, due to their conservation status, account for 36 species, belonging to 26 families and eight orders (Table 2). Twenty-eight species are in IUCN Red List under threatened categories (Vulnerable, Endangered and Critically Endangered), while seven species are in the Near Threatened category. Nothing from recent species in the collection was considered Extinct (EX). Excluding a bovid species, the Grey rhebok *Peleacapreolus* (NT category by IUCN), all other species (*n* = 35) are included in one or more appendices of CITES. It was not possible identify at the species level the primate specimens of the genus *Tarsius* (*n* = 2). However, this genus includes 11 species under some IUCN threatened category and one species as Data Deficient, while all species are included in CITES Appendix II.

The most ancient specimens date back to the early 17^th^ century (ca. 1600-1620) and belong to the Kircherian collection. This material consists of fifteen specimens, including anatomical pieces from three species of Rhinocerontidae and one walrus, *Odobenusrosmarus*, all of them under some threatened category.

## Discussion

This catalogue represents the status of the medium and large-sized terrestrial mammals present at the Museum of Comparative Anatomy “Giovanni Battista Grassi” in early 2024. Currently, 27 orders of recent mammals are recognised (Burgin et al. 2018), five them not being considered in the present study, but are represented in the collection: Didelphimorphia, Rodentia, Eulipotyphla, Lagomorpha and Chiroptera. Considering only the categories studied here, 63% of recent mammalian orders were identified, while the orders Paucituberculata, Microbiotheria, Notoryctemorphia, Peramelemorphia, Tubulidentada and Afrosoricida are not represented.

The collection includes specimens from all major biogeographical regions. For example, there are specimens from the "Wallacea", organised in a specific display case (including *Babyrousacelebensis*, *Tarsius* sp. and *Pongopygmaeus*) and from the Neotropical Region, already widely discussed in Gippoliti and Castiglia (2020). The collection also includes historical specimens derived from Italian expeditions in Africa during the early 20^th^ century (e.g. Primates - Bruner and Gippoliti (2006)).

### Museomic and specimens of special interest

Despite the lack of data on the collection location in numerous specimens, the mammal collection at the Museum constitutes an important source for various research areas focused on the functional and evolutionary morphology of vertebrates, as well as on molecular genetics. Besides taxonomic and systematic studies, species conservation could greatly benefit from biodiversity genomics approaches (Ernst et al. 2022), while specimens from ancient or extinct populations can help to understand extinction processes and genetic diversity (Cilli et al. 2023, Theissinger et al. 2023).

The Museum collections have already been used in studies on ursids (Meloro et al. 2017), rhinocerotids (Pandolfi et al. 2019), suids (Iannucci et al. 2020, Iannucci et al. 2022), canids (Cerilli and Fatucci 2021), and hippos (Caloi et al. 1980, Mecozzi et al. 2023). Furthermore, in times of ‘museomics’ (Mandrioli 2016), having access to historical specimens is advantageous for studies on taxonomy, systematics and population and landscape genetics (e.g. Bi et al. 2013, Abreu-Jr et al. 2020, Giarla and Voss 2020, Roycroft et al. 2021), including samples from the Museum collections (Pasino et al. 2023).

It is essential to mention the presence of an entire skeleton of *Hippopotamusamphibius*, already discussed by Di Russo et al. (2024) and here with complementary data. This specimen (catalogue number ac0193) comes from the Archiginnasio collection and was catalogued by Metaxà (1853) with the notation on a label “«Esemplare dell’Egitto. Dono dalla S.M. Gregorio XVI, che ebbe da Clot-Bey, medico del viceré d’Egitto»” (translating as “Specimen of Egypt. Gift from the P.M. Gregory XVI, which he received from Clot-Bey, doctor of the viceroy of Egypt"). Pope Gregory XVI pontificated during 1831-1846, while the French doctor Antoine Clot-Bey arrived in Egypt in early 1825 (Burrow 1975) and published a monograph on Egypt with personal comments 15 years later (Clot-Bey 1840). In his monograph, Clot-Bey comments that hippopotamus individuals were found along the high Nubie River, in Nubia, where the collector Prosper Alpin told him he had hunted an individual (Clot-Bey 1840, page 136). Thus, it is the probable period of collection of the specimen, between 1825 and 1840 and it was collected along the high Nubie River. This specimen has also been studied for its morphological characters of the skull and postcranium skeleton (Caloi et al. 1980) and could be the subject of future studies, including molecular ones.

Of special interest are also the specimens from the Kircherian collection, dating from the early 17^th^ century. This collection consists of 15 specimens and samples (Bonanni 1709, Anonymous 2015) and includes a hippopotamus specimen and all eight rhinoceros specimens housed in the Museum. These species are under some threatened category and international trade regulation (IUCN 2023, CITES – Convention on International Trade in Endangered Species of Wild Fauna and Flora 2023) and it is probable that the specimens from this collection are derived from extinct populations.

## Conclusions

Important outcomes can derive from this study, both in scientific scope and human resources formation. By disclosing the holdings of the historical mammal collections of the Museum, we hope that the international academic community be made aware of these representative specimens of extirpated populations of threatened species. Another key is the enrolment of undergraduate students in scientific areas, towards which few of them move, such as morphology, taxonomy and scientific collections (Carvalho et al. 2005, Pearson et al. 2011). Together, scientific disclosure and training students can shed light on biological scientific collections and generate interest in them, helping to minimise the progressive loss of scientific relevance of Italian natural history collections Andreone et al. 2014, Andreone et al. 2022

## Supplementary Material

664D7543-388C-5674-8381-112143A81ED8 10.3897/BDJ.12.e124810.suppl1 Supplementary material 1 Medium and large-sized terrestrial mammal specimens at the Museo di Anatomia Comparata “Giovanni Battista Grassi” Data type Collection database Brief description Excel database of the specimens, including voucher numbers, taxa levels up to species level, Conservations status, sex, Anatomic structure, Preservation type, Collection date, Country, Locality and Ecoregion. File: oo_1020133.xlsx https://binary.pensoft.net/file/1020133 Alexandra M.R Bezerra, Edoardo Di Russo and Riccardo Castiglia

## Figures and Tables

**Table 1.** Family-level account of medium and large-sized terrestrial mammal specimens at the Museo di Anatomia Comparata “Giovanni Battista Grassi”. Skin - includes stuffed and open skin preparations; Skull – not including detached horns and antlers; Skeleton – only skull + postcranium skeletons; Bones - including any detached bone and keratin appendices; Spirits – 70% ethanol or formaldehyde. *N* = number of specimens per family, asterisk (*) = teeth samples, either in Fossil or Bones columns. Obs: 1. One Falange and one claw of extinct ground sloths; 2. Burgin et al. (2018) include Callitrichidae under Cebidae; here we separated for didactic proposal; 3. Includes a formalin-fixed and dry-preserved encephalic.

Taxon	Genera	Species	Fossil	Skin	Skull	Skeleton	Bones	Spirits	*N*
**Class Mammalia**									
Subclass Prototheria									
**Order Monotremata**									
Family Ornithorhynchidae	1	1		1		2			3
Family Tachyglossidae	1	1		1		1			2
Subclass Theria									
Infraclass Marsupialia									
**Order Dasyuromorphia**									
Family Dasyuridae	1	1		1					1
**Order Diprotodontia**									
Family Macropodidae	3	5		1	3	1			5
Not identified	-	-		1					1
Family Phalangeridae	1	1			1				1
Family Phascolarctidae	1	1		1					1
Infraclass Placentalia									
Superorder Afrotheria									
**Order Hyracoidea**									
Family Procaviidae	1	2		1	2	2			5
**Order Macroscelidea**									
Family Macroscelididae	1	1			1				1
**Order Proboscidea**									
Family Elephantidae	3	4	5*				1*		6
Not identified	-	-	16*						16
**Order Sirenia**									
Family Dugongidae	1	1			1				1
Superorder Xenarthra									
**Order Cingulata**									
Family Dasypodidae	3	3		2	2	2			6
**Order Pilosa**									
Family Bradypodidae	2	2		1		1			2
Family Megatheriidae	1	1	2^1^						2
Family Myrmecophagidae	2	3		1	1	1			3
Superorder Euarchontoglires									
**Order Dermoptera**									
Family Cynocephalidae	1	1		1					1
**Order Primates**									
Not identified	-	-				2	6	1	9
Family Atelidae	2	2		1	1	1			3
Family Callithrichidae^2^	1	1		1	1				2
Family Cebidae	2	2			2	1		1	4
Family Cercopithecidae	6	7		2	8	7	2	3	22
Not identified	-	-			1	1			2
Family Daubentoniidae	1	1				1			1
Family Galagidae	1	1		1					1
Family Hominidae	4	4		1	13	5	10^3^	12	41
Family Hylobatidae	1	1		1					1
Family Lemuridae	3	3		1	3				4
Family Lorisidae	2	2			2	1			3
Family Tarsiidae	1	2		1		1			2
**Order Scandentia**									
Family Tupaiidae	1	1			1				1
Superorder Laurasiatheria									
**Order Artiodactyla**									
Not identified	-	-			1		3		4
Family Bovidae	13	19		1	23	9	17	4	54
Not identified	-	-			1		4		5
Family Camelidae	2	2			2	1		1	4
Not identified	-	-			1				1
Family Cervidae	3	5	1*	1	5		9		16
Not identified	-	-					1		1
Family Giraffidae	1	1				1		2	3
Family Hippopotamidae	1	2			1	1	2*		4
Family Suidae	2	2			4	1	3	3	11
Not identified	-	-					2		2
Family Tayassuidae	1	1		1	1				2
Family Tragulidae	1	1		1					1
**Order Carnivora**									
Not identified	-	-				2	1	1	4
Family Canidae	2	6		2	45	3	3	5	58
Not identified	-	-			1				1
Family Felidae	4	9		1	21	7	1	6	36
Not identified	-	-			2	2			4
Family Herpestidae	3	3		1	3				4
Family Hyaenidae	2	2			4	1			5
Family Mephitidae	1	1			1				1
Family Mustelidae	4	7		7	5	4		1	17
Family Odobenidae	1	1					1		1
Family Phocidae	2	2		1	2	1			4
Family Procyonidae	2	2		1	1				2
Family Ursidae	1	2			4	2			6
Family Viverridae	2	4		1	6	1			8
**Order Perissodactyla**									
Not identified	-	-			1				1
Family Equidae	1	2	1*		5	2	10	1	19
Not identified	-	-			1	1			2
Family Rhinocerotidae	3	3			1		6		7
Family Tapiridae	1	1			1				1
**Order Pholidota**									
Family Manidae	1	1		1		1			2
Total	101	133	25	40	186	70	82	41	444

**Table 2.** Threatened species at the Museo di Anatomia Comparata “Giovanni Battista Grassi”. IUCN categories: NT – Near Threatened, Vulnerable – VU, Endangered – EN, Critically Endangered – CR. CITES Appendices I, II and III. Including biographic realm origin and habitat (terrestrial or aquatic) and total of specimens (Total). Obs: 1. The genus *Tarsius* includes 11 species under some IUCN threatened category and one species as Data Deficient, while all species are included in the CITES Appendix II.

Taxon	IUCN	CITES	Biographic Realm	Habitat	Total
**Order Cingulata**					
Myrmecophagidae					
*Myrmecophagatridactyla* Linnaeus, 1758	VU	II	Neotropics	Terrestrial	1
**Order Proboscidea**					
Elephantidae					
*Loxodontaafricana* (Blumenbach, 1797)	EN	II	Afrotropic	Terrestrial	1
**Order Sirenia**					
Dugongidae					
*Dugongdugon* (Müller, 1776)	VU	I	Afrotropic, Indo-Malay, Australasia	Marine	1
**Order Primates**					
Atelidae					
*Alouattapalliata* (Gray, 1849)	VU	I	Neotropics	Terrestrial	2
Cercopithecidae					
*Erythrocebuspatas* (Schreber, 1774)	NT	II	Afrotropic	Terrestrial	1
*Macacasylvanus* (Linnaeus, 1758)	EN	I	Palearctic	Terrestrial	2
Daubentoniidae					
*Daubentoniamadagascariensis* (Gmelin, 1788)	EN	I	Afrotropic	Terrestrial	1
Galagidae					
*Sciurocheirusalleni* (Waterhouse, 1838)	NT	II	Afrotropic	Terrestrial	1
Hominidae					
*Gorillagorilla* (Savage, 1847)	CR	I	Afrotropic	Terrestrial	1
*Pantroglodytes* (Blumenbach, 1799)	EN	I	Afrotropic	Terrestrial	3
*Pongopygmaeus* (Linnaeus, 1760)	CR	I	Indo-Malay	Terrestrial	2
Hylobatidae					
*Symphalangussyndactylus* (Raffles, 1821)	EN	I	Indo-Malay	Terrestrial	1
Lemuridae					
*Eulemurfulvus* (É. Geoffroy, 1796)	VU	I	Afrotropic	Terrestrial	1
*Vareciavariegata* (Kerr, 1792)	CR	I	Afrotropic	Terrestrial	1
Lorisidae					
*Loristardigradus* (Linnaeus, 1758)	EN	II	Indo-Malay	Terrestrial	1
*Perodicticuspotto* (Müller, 1766)	NT	II	Afrotropic	Terrestrial	2
Tarsiidae					
*Tarsius* sp.	?^1^	II	Indo-Malay	Terrestrial	2
**Order Artiodactyla**					
Bovidae					
*Addaxnasomaculatus* (de Blainville, 1816)	CR	I	Afrotropic	Terrestrial	1
*Gazelladorcas* (Linnaeus, 1758)	VU	III	Palearctic	Terrestrial	1
*Peleacapreolus* (Forster, 1790)	NT	-	Afrotropic	Terrestrial	1
Giraffidae					
*Giraffacamelopardalis* (Linnaeus, 1758)	VU	II	Afrotropic	Terrestrial	3
Hippopotamidae					
*Hippopotamusamphibius* Linnaeus, 1758	VU	II, III	Afrotropic	Terrestrial, Freshwater, Marine	3
Suidae					
*Babyrousacelebensis* (Daninger, 1909)	VU	I	Indo-Malay	Terrestrial	1
**Order Carnivora**					
Felidae					
*Acinonyxjubatus* (Schrebr, 1775)	VU	I	Afrotropic, Palearctic	Terrestrial	3
*Pantheraleo* (Linnaeus, 1758)	VU	I, II, III	Afrotropic	Terrestrial	6
*Pantherapardus* (Linnaeus, 1758)	VU	I	Afrotropic, Palearctic, Indo-Malay	Terrestrial	4
*Pantheratigris* (Linnaeus, 1758)	EN	I, II	Indo-Malay	Terrestrial	2
Hyaenidae					
*Hyaenahyaena* (Linnaeus, 1758)	NT	III	Afrotropic, Palearctic, Indo-Malay	Terrestrial	3
Mustelidae					
*Lutralutra* (Linnaeus, 1758)	NT	I, III	Palearctic, Indo-Malay	Terrestrial, Freshwater, Marine	1
Odobenidae					
*Odobenusrosmarus* (Linnaeus, 1758)	VU	III	Palearctic, Nearctic	Terrestrial, Marine	1
Phocidae					
*Monachusmonachus* (Hermann, 1779)	EN	I	Palearctic	Terrestrial, Marine	1
Ursidae					
*Ursusmaritimus* Phipps, 1774	VU	II, III	Palearctic, Nearctic	Terrestrial, Marine	2
**Order Perissodactyla**					
Rhinocerotidae					
*Ceratotheriumsimum* (Burchell, 817)	NT	I, II	Afrotropical	Terrestrial	1
*Dicerosbicornis* (Linnaeus, 1758)	CR	I, II	Afrotropical	Terrestrial	5
Rhinoceroscf.unicornis	VU	I	Indo-Malay	Terrestrial	1
**Order Pholidota**					
Manidae					
*Manisjavanica* Desmarest, 1822	CR	I, II	Indo-Malay	Terrestrial	2
Total					66
